# Smoking, alcohol and opioids effect on coronary microcirculation: an update overview

**DOI:** 10.1186/s12872-021-01990-y

**Published:** 2021-04-15

**Authors:** Zahra Jalali, Morteza Khademalhosseini, Narjes Soltani, Ali Esmaeili Nadimi

**Affiliations:** 1 Non-Communicable Diseases Research Center, Rafsanjan University of Medical Sciences, Building Number 1, Emam Ali Boulevard, P.O. Box: 77175-835, 7719617996 Rafsanjan, Iran; 2 Department of Clinical Biochemistry, School of Medicine, Rafsanjan University of Medical Sciences, Rafsanjan, Iran; 3 Department of Pathology, Rafsanjan University of Medical Sciences, Rafsanjan, Iran; 4 Department of Cardiology, School of Medicine, Rafsanjani University of Medical Sciences, Rafsanjan, Iran

**Keywords:** Smoking, Alcohol, Opioid, Coronary microvascular dysfunction, Ischemic heart disease, Myocardial infarction

## Abstract

Smoking, heavy alcohol drinking and drug abuse are detrimental lifestyle factors leading to loss of million years of healthy life annually. One of the major health complications caused by these substances is the development of cardiovascular diseases (CVD), which accounts for a significant proportion of substance-induced death. Smoking and excessive alcohol consumption are related to the higher risk of acute myocardial infarction. Similarly, opioid addiction, as one of the most commonly used substances worldwide, is associated with cardiac events such as ischemia and myocardial infarction (MI). As supported by many studies, coronary artery disease (CAD) is considered as a major cause for substance-induced cardiac events. Nonetheless, over the last three decades, a growing body of evidence indicates that a significant proportion of substance-induced cardiac ischemia or MI cases, do not manifest any signs of CAD. In the absence of CAD, the coronary microvascular dysfunction is believed to be the main underlying reason for CVD. To date, comprehensive literature reviews have been published on the clinicopathology of CAD caused by smoking and opioids, as well as macrovascular pathological features of the alcoholic cardiomyopathy. However, to the best of our knowledge there is no review article about the impact of these substances on the coronary microvascular network. Therefore, the present review will focus on the current understanding of the pathophysiological alterations in the coronary microcirculation triggered by smoking, alcohol and opioids.

## Background

Different studies have shown that 20% to 50% of angina patients undergoing coronary angiography have normal or near normal arteries, (non-obstructive coronary disease) [1–9]. These groups of patients were conventionally diagnosed as Cardiac Syndrome X. Today it is demonstrated that a large proportion of these patients have coronary microvascular dysfunction (CMD) [3]. CMD has gained more attention over the last 15 years as a cardiac cause of morbidity and as critical as CAD. CMD diagnosed patients display high rates of hospitalization for unstable angina, myocardial infarction (MI) and heart failure [7, 10, 11]. Pathophysiology studies suggest that CMD develops by: (1) structural alterations including the remodeling of microvessels or microcircular rarefaction; or (2) functional abnormalities such as spasm or impaired dilatory function in endothelial or vascular smooth muscle cell (VSMC). The underlying mechanisms are the dysregulation of the related hormonal, metabolic or neurosympathetic (neural tone) stimuli [12–14]. CMD can occur as a primary condition in patients with no obstructive coronary disease or can exist in the setting of diffuse and focal epicardial coronary disease [15] or occur as a consequence of acute MI or other cardiac events leading to damage in coronary microcirculation (e.g. percutaneous coronary intervention). In case CMD and epicardial problems exists side by side, the diagnosis of CMD is challenging but clinically important for determining the prognosis of ischemic and angina patients. For example, diagnosis of CMD can explain why symptoms persist in some CAD patients following percutaneous coronary interventions (PCI) [15–17]. CMD diagnosis is often challenging and not as well established as diagnosis of CAD. Yet no imaging method is available to visualize vessels smaller than 500 µm directly. Therefore, only indirect measurements of microvascular function are practiced in clinical settings today. Commonly, coronary flow reserve (CFR) is used as an indirect indicator of coronary microvascular function in the absence of CAD (normal or near normal angiography) [18–22]. CFR represents the capacity of coronary circulation to increase coronary blood flow (CBF) from basal levels to maximum in response to a vasodilatory stimulus. Therefore, CFR is proportional to blood flow in epicardial vessels plus microvasculature [23]. The reduced coronary flow reserve (CFR) manifested in CMD patients with no obstructive CAD (determined by normal angiography), is due to an impaired coronary vasodilation of microvasculature at higher demanding conditions of myocardium [23, 24].

Today, coronary microvascular function can be measured using invasive angiographic methods such as Doppler-tipped coronary guidewire and wire-based thermodilution techniques. Additionally, non-invasive imaging technologies such as positron emission tomography (PET) cardiac magnetic resonance imaging (CMR) or transthoracic Doppler echocardiography of the left anterior descending coronary artery are applied to measure indexes of coronary microvascular function. Therefore, one complexity for diagnosis of CMD is that it requires technologies that may not be widely available [14, 25–27]. CMD is associated with conventional cardiac risk factors such as smoking, aging, obesity, diabetes mellitus, and hypertension [18, 28]. However, growing number of publications point to the significant contribution of non-traditional risk factors in CMD development, including substance abuse (e.g., alcohol, opioids, cocaine). Here, we overview and highlight on the clinical and pathophysiological effects of substance abuse i.e., smoking, alcohol and opioids on CMD, with an aim to bring this topic to the attention of more researchers in the field.

### Search strategy

In this literature review, a systematic search strategy was performed in electronic scientific databases PUBMED, Medline, Google Scholar using advanced search and all combinations of search terms. Search terms were selected based on the entry terms suggested by Medical Subject Headings (MeSH). Title and abstracts were first screened based on relevance to the subject and the study was selected to be included in the review after assessing the full text for eligibility and relevance. Additionally, the reference and citing publication lists from the retrieved articles were checked to identify further relevant studies. The search time limit was since 1980 to date. We included only studies published in English. Our search terms include: (heart, coronary); (percutaneous coronary intervention, percutaneous coronary, revascularization, reperfusion, angioplasty); (smoking, tobacco, cigars, cigarette); (microvessel, Microvasculature, microvascular network, microvascular, microcirculation, small vessels, capillary, arteriole, angiogenesis, intimal proliferation, intimal neoplasia, neointimal, microvascular obstruction, MVO, coronary flow reserve, CFR, index of microvasculatory resistance, IMR); (Ethanol, alcohol, ethyl, EtOH, alcoholic, drinking, wine); (opioid, opiate, opium, morphine, amphetamine, methadone, analgesics) and (ischemia, infarction, MI).

## Impact of smoking on coronary microvasculature

### Impact of smoking on coronary microvasculature and stable CMD

#### Smoking pattern impact on the coronary microvascular function

Epidemiological and case–control studies demonstrated chronic long-term smoking as one of the predictors of CMD in asymptomatic individuals [29–32] the non-obstructive coronary ischemic patients (no CAD) (stable CMD) [33, 34] as well as patients with vasospastic Angina [35] or CAD [36] background. These studies rely on the measured CFR and its stimulus-induced changes, due to unavailability of a direct technique to assess the coronary microvasculature status in vivo. Compared to long-term effects, the short-term chronic smoking assessed in healthy young smokers (with no evidence of CAD) did not affect the myocardial blood flow at resting conditions. However, smokers displayed a lower CFR in response to stress [29, 31].

Acute smoking can also exert negative impact on coronary microvascular function (in habitual smokers or non-smokers). Park et al. conducted a study on healthy young smokers and non-smokers, comparing CFR and the coronary vascular resistance index (CVRI) after a 4-h period of smoking abstinence. No significant difference between smokers and non-smokers was observed. However, after consumption of only two cigarettes in the smoking group, a considerable decline in CFR and an increase in CVRI were observed in the smokers [37].

On the other hand, the acute CFR declining effect was shown to be equivalent upon light (containing 0.6 mg nicotine, 8 mg tar, 9 mg carbon monoxide) and regular cigarette smoking (containing 0.9 mg nicotine, = 12 mg tar, 12 mg carbon monoxide) [38, 39]. On the contrary, one group found enhanced acute effect of high nicotine content cigarettes on CFR compared to the low content ones, showing a dose-dependent contribution of nicotine component of cigarettes in microvascular damaging effects of smoking [40].

Multiple studies have implicated passive smoking as a significant risk factor in CHD, being associated with higher rate of morbidity and mortality and poor outcome in CHD and acute coronary syndrome patients [41–49]. Additionally, an impairment of microvascular function and reactivity was supported by other studies indicating passive smoke exposure as a risk factor in CMD development [50, 51]. On the other hand, passive smoking is associated with lower odds ratio of smoking cessation in CHD patients [52, 53]. This is important due to the fact that smoking cessation is considered as a major preventive measure and management strategy to reduce mortality risk among CHD patients [54, 55] as well as patients with coronary microvascular dysfunction [56, 57].

Based on current information on the effects of different patterns of smoking on CMD (light vs. regular cigarette; active vs. passive smoking), evidence does not support one pattern over the other, emphasizing on the detrimental effects of smoking nevertheless. However, the limited number of studies on smoking-connected CMD, and particularly the small population size in most of these studies, necessitates conduction of large-scale research on this subject in the future.

#### Mechanistic studies on the effects of smoking on coronary microvasculature in human subjects

**Fig. 1:**
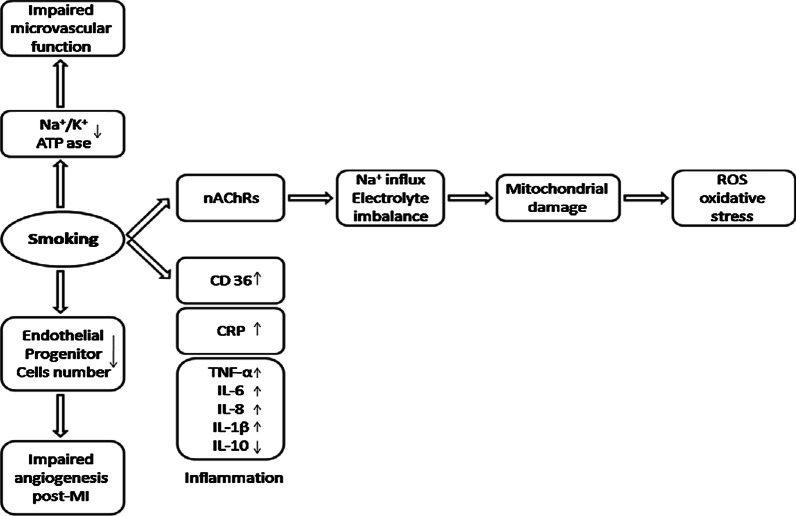
Pathophysiology of smoking-triggered coronary microvascular damage. Summary of the underlying mechanisms of coronary microvascular damage caused by smoking

Evidence indicates that smoking affects coronary microvasculature via altering endothelial cells. Impaired coronary microvascular function in healthy young chronic smokers was observed under cold stress as an endothelium-dependent stimuli [29, 31] whereas dipyridamole which acts through endothelium-independent mechanisms, failed to affect microvascular function in healthy smokers [29]. The main mechanisms underlined in the literature for the smoking-induced vascular endothelial cell damage are oxidative stress, inflammation, impaired Na^+^/K^+^ ATPase function (Fig. 1). Cigarette smoke contains several radical or non-radical oxidants including superoxide radicals (^.^O2−), hydroxyl radicals (^.^OH), and peroxides (ROOH). Therefore, smoking can induce oxidative stress directly via its content and cause damage to coronary microvascular cells [58, 59]. It was shown that vitamin C as a potent antioxidant has been demonstrated to normalize the impaired coronary microvascular function in chronic smokers, whereas it did not alter CFR in non-smokers at all [32]. In addition to the oxidative stress, smoking is considered as a potent pro-inflammatory factor in cardiac pathology. The reciprocal stimulatory crosstalk between inflammation and oxidative stress has been widely discussed in the literature [60].

Multiple studies have linked chronic inflammatory disorders such as lupus, rheumatoid arthritis, and inflammatory bowel disease with higher risk of developing CMD [61–63]. Others confirmed this result finding a significant correlation between systemic inflammatory markers (mostly plasma CRP) and risk of CMD [64–66]. Recently, Schroder et al. assessed the differential blood protein profile of CMD patients and healthy volunteers. Interestingly, the main differentially expressed protein biomarkers they found to be associated with CMD were several pro-inflammatory factors, related to the TNF-α-IL-6-CRP pathway [66]. Smoking is conventionally considered to induce inflammation based on studies who rely on self-reports on smoking status [67–70]. Recently, the National Health Survey in Korea conducted on 8655 men and 10,432 women reported a systemic pro-inflammatory effect of smoking. This study investigated the dose-dependent effect of cigarette smoking based on the cotinine concentration in the urine, on the systemic inflammation measured by leukocyte count [71]. In addition, they found higher plasma levels of inflammatory markers TNF-α, IL-6, IL-8 and IL-1β in current smokers, while the anti-inflammatory marker IL-10 showed reduction [58].

Rooks et al. measured CFR and different inflammatory markers (e.g., interleukin-6 and C-reactive protein), and oxidative status indicators (e.g., plasma hydroperoxides and the glutathione oxidation (GSSG/GSH ratio)) in healthy smoker and non-smoker twin couples. After adjusting the CFR levels to the inflammatory and oxidative stress indices, the declined level of CFR in smokers retained; which implicates the contribution of other underlying mechanisms to this difference, other than inflammation and ROS [30]. One such mechanism may be smoking-induced downregulation of ATPases [72]. Impaired microvascular reactivity and vasodilation in chronic smokers have been suggested to be caused at least partially because of the down-regulation of Na^+^/K^+^ ATPase in coronary microcirculatory endothelial cells in smokers. Since, Quabain as an inhibitor of Na^+^/K^+^ ATPase, that normally induces vasodilation in coronary microcirculation, failed to act on chronic smokers' microcirculation [72].

#### Mechanistic ex vivo and in vitro evidence on the effects of nicotine on coronary microvasculature

Nicotinic acetylcholine receptors (nAChRs) are shown to express in coronary endothelial cells, and induce several pro-survival pathways upon normal physiologic stimulation [73]. However, their overstimulation in cells by chronic nicotine exposure causes aberrant microvascular dilatory functions via oxidative stress induction [74]. Induction of oxidative stress by overstimulation of nicotinic AChRs is due to their intrinsic cationic channel function [75]. Chronic nicotine exposure induces opening of these channels and an excessive influx of Na^+^ ions in to the cells. The following electrolyte imbalance results in overproduction of ROS and consequently cellular damage [76]. The effect of nicotine tested in vivo (rat model) demonstrated an impaired endothelial-dependent vascular function, concurrent with plasma increased level of inflammatory parameter CRP, as well as higher CD36, TNFα and IL1β in macrophages [77]. The pro-inflammatory effect of smoking and nicotine are on the other hand contradicted by findings of other in vivo and in vitro studies reporting that nicotine affects vascular endothelial cells and macrophages by reducing their production of inflammatory cytokines (e.g. TNF- α), and consequently their capacity for leukocyte recruitment and adhesion [78, 79]. The reason for the contradictory results regarding the pro- or anti-inflammatory impact of nicotine is not understood yet, and warrants further assessment in the future.

### Impact of smoking on post-ischemic and PCI-induced coronary microvascular injury

#### Clinical studies on the impact of smoking on coronary microvasculature injury by reperfused myocardial infarction

Microvascular dysfunction as either microvascular intraluminal obstruction (MVO) or extravascular compression is one of the main non-reversible consequences of coronary ischemic reperfusion injury (IRI) caused by cardiac therapeutic interventions [80, 81]. Ischemia causes mitochondrial damage, plasma membrane and cytoskeleton disintegrity, and impaired enzymatic activities in the cells by lowering the pH and ATP levels, inducing dysfunction of the ion exchange factors and electrolyte imbalance. Upon reperfusion and reversal of oxygen supply, the impaired ion exchangers, enzymes, cellular membrane and mitochondria result in reciprocal enhancement of oxidative stress and inflammation that may result in severe cell damage or cell death [82]. Therefore, knowing the risk factors that exacerbate this problem or on the contrary preconditioning or therapeutic mechanisms, which protect against the excessive oxidative damage, inflammation and cell death in post-MI conditions or by IRI are currently under special attention of researchers and clinicians. Although cigarette smoking is an independent risk factor for cardiovascular disease, studies have reported controversial results on the effects of smoking on the mortality rate and prognosis of patients who underwent reperfusion with a percutaneous coronary intervention (PCI) or the extent of post-MI injury (smoker’s paradox phenomenon) which are previously reviewed elsewhere [55, 83–85]. Here we review studies which assessed the status of coronary microvasculature in these group of patients. Based on magnetic resonance (CMR) analysis in reperfused ST-segment elevation myocardial infarction (STEMI) patients, smoking displayed no significant association with microvascular obstruction (MVO) [86, 87] and index of microvascular resistance (IMR) assessed by intra-coronary sensor angiography [88].

However, smoking was associated with intramyocardial haemorrhage (IMH) in post-PCI STEMI patients [86, 88] and in the presence of IMH, the protective effect of smoking on post-PCI cardiovascular health was abolished suggesting higher microvascular injury by post-ischemic reperfusion in smokers [86]. IMH is a marker of severe and irreversible injury to the coronary microvasculature by ischemia–reperfusion which results to extravasation of erythrocytes [89].

This result is challenged by another study using CMR is reperfused STEMI patients in another population, which did not find a significant association between smoking and the frequency of IMH [90]. The effect of smoking on endothelial progenitor cells (EPC) as necessary factors for repair and regeneration of microvasculature post-ischemia [91] was investigated by several studies. Smoking is shown to reduce the number of EPC, their adhesive capacity, and colony forming abilities [56, 91–93]. Therefore, if the damage to the coronary microcirculation is not irreversible (IMH), smoking may have beneficial impact on coronary microvascular regeneration post ischemia and reperfusion. Further research is warranted to assess this phenomenon in the future.

#### Ex vivo and in vitro effects of smoking on post ischemic and PCI-induced microvascular injury

Animal studies indicate that nicotine has a pathologic angiogenic effect on coronary arteries and microvessels, and intimal hyperplasia post ischemia and in PCI [94, 95]. Nicotine angiogenic effects are mediated by nicotinic acetylcholine receptors in endothelial cells [95–97]. Neointimal formation induced by nicotine effect on VSMC post injury has been linked to ERK –Egr-1 signaling cascade, and the blockade of this pathway can revert the adverse effect of nicotine in coronary vascular remodeling [98].

## Impact of alcohol on coronary microvasculature

### Impact of alcohol on coronary microvasculature and stable CMD

#### Clinical impact of alcohol on the coronary microvascular function

Detrimental health effects of alcohol include cardiometabolic complications which account for 33% of death caused by alcohol [99]. Multiple studies demonstrated regular and irregular heavy drinking to markedly increase the risk of ischemic heart disease and hypertension [100–102]. However, the general effect of alcohol consumption on cardiovascular disease is considered to be complicated due to other reports which support a protective role for the low and moderate alcohol drinking in regard to the ischemic heart disease and MI [103, 104]. In this section we will review the current understanding of the impact of alcohol on coronary micro-vessels. Similar acute effect was observed for two moderate doses of red wine (not vodka or white wine) to improve CFR in healthy young individuals, indicating a vasodilatory and cardioprotective function. These doses of red wine correspond the amount of 0.5 and 1.0 g/kg ethanol. The level of CFR increase was correlated with the level of antioxidant capacity of plasma induced by alcohol containing red wine. Improtantly, de-alcoholized red wine had no such effects. The CFR was measured by transthoracic Doppler echocardiography and right after bevarage drinking (acute effect of ethanol) [105]. This was contradicted by another study that measured CFR (by myocardial contrast echocardiography) in response to 1–2 weeks consumption of moderate dose of ethanol (red wine) and observed no change in CFR. The difference could be due to the fact that in the later study measurements were made at least 12 h after alcohol consumption; therefore, it cannot represent the acute effects of alcohol consumption, compared to the abovementioned studies which assessed CFR right after drinking [106].

#### Mechanistic studies on the effects of alcohol on coronary microvasculature in human subjects

**Fig. 2:**
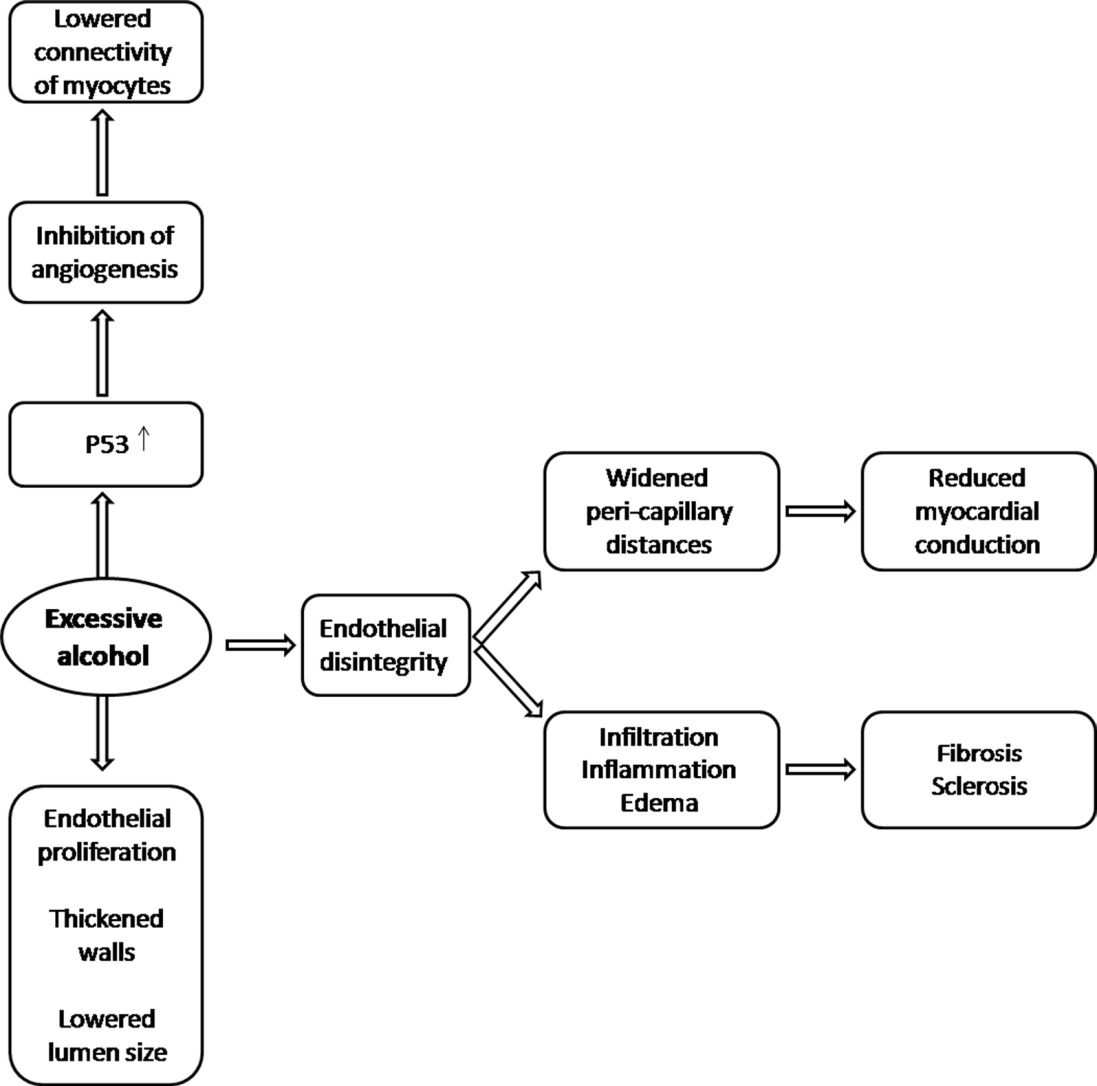
Pathophysiology of heavy alcohol-mediated coronary microvascular damage. Summary of the underlying mechanisms of coronary microvascular damage caused by heavy alcohol drinking

Heavy alcohol has been shown to result in deleterious remodeling and ultra-structural alterations of the cardiac microcirculation, depicted by case–control studies that used histochemical staining, and microscopy on cardiac biopsies obtained from alcoholic patients (angina patients with no CAD) after their death. Briefly in regard to the underlying pathophysiological mechanisms of alcohol-induced damage, the results showed disorganization of the layers of the micro-vessel walls, edema, perivascular fibrosis, sclerosis, interstitial inflammation, the degeneration of endothelial cells and higher density of capillary network [107, 108] (Fig. 2).

#### Mechanistic ex vivo and in vitro evidence on the effects of alcohol on coronary microvasculature

Animal studies indicate increased coronary microvascular wall thickness and an enrichment of the ATP-hydrolyzing small-caliber micorvessels [109]. Morphometric analysis by electron microscopy confirmed structural changes in the endothelial cells of the capillaries but not the muscle cells. Numerical density of endothelial cells was enhanced, whereas the volume density did not show a significant alteration, indicative of the proliferation of endothelial cells [110].

Others reported widened peri-capillary distances resulting in enforced remodeling changes in the size and connectivity of cardiomyocytes, and subsequently impaired myocardial conduction in animal models [111]. Evidence suggests that alcohol-induced hypoxia-mimetic and metabolically demanding condition [112] in the endothelial cells, lead to the endothelial remodeling and degeneration. Consequently, the damage of the micro-vessel endothelial cells precipitates in an increased infiltration of fluids and metabolites to the vessel walls and the perivascular space, which in turn results in edema and inflammation [111, 113]. This mechanism consequently induces the reported deposit of higher levels of collagen, perivascular fibrosis and sclerosis, and declined conductivity [111].

### Impact of alcohol on post-ischemic and PCI-induced coronary microvascular injury

#### Clinical studies on the impact of alcohol on coronary microvasculature injury by reperfused myocardial infarction

Studies have shown better prognosis and lower mortality rate post MI [114–117] upon prior moderate but not heavy chronic alcohol consumption. In regard to PCI however, administration of a moderate dose of ethanol displayed an adverse effect on myocardial ischemic damage post PCI in STEMI patients [118]. Specific impact of moderate and heavy alcohol consumption on coronary microvascular function remains unfeatured as yet, warranting studies that assess the corresponding indices (e.g., CFR, MVO and IMR) in relation to beverage type, pattern and duration of alcohol consumption in MI patients.

#### Ex vivo and in vitro effects of alcohol on post ischemic and PCI-induced microvascular injury

The angiogenesis occurs after myocardial injuries such as ischemia to provide oxygen and supplies for the regeneration process of the myocardium. In vivo study in rat model preconditioned with either a high, or a modest dose of alcohol before the induction of MI, showed that the angiogenesis is significantly increased upon moderate alcohol preconditioning; whereas it was reduced in excessive dose of alcohol consumption prior to MI [119]. The level of VEGF expression did not change upon intake of high ethanol doses, while endostatin was upregulated. Conversely, upon moderate alcohol intake, VEGF level was shown to be up-regulated, whereas endostatin expression significantly declined [119].

**Fig. 3:**
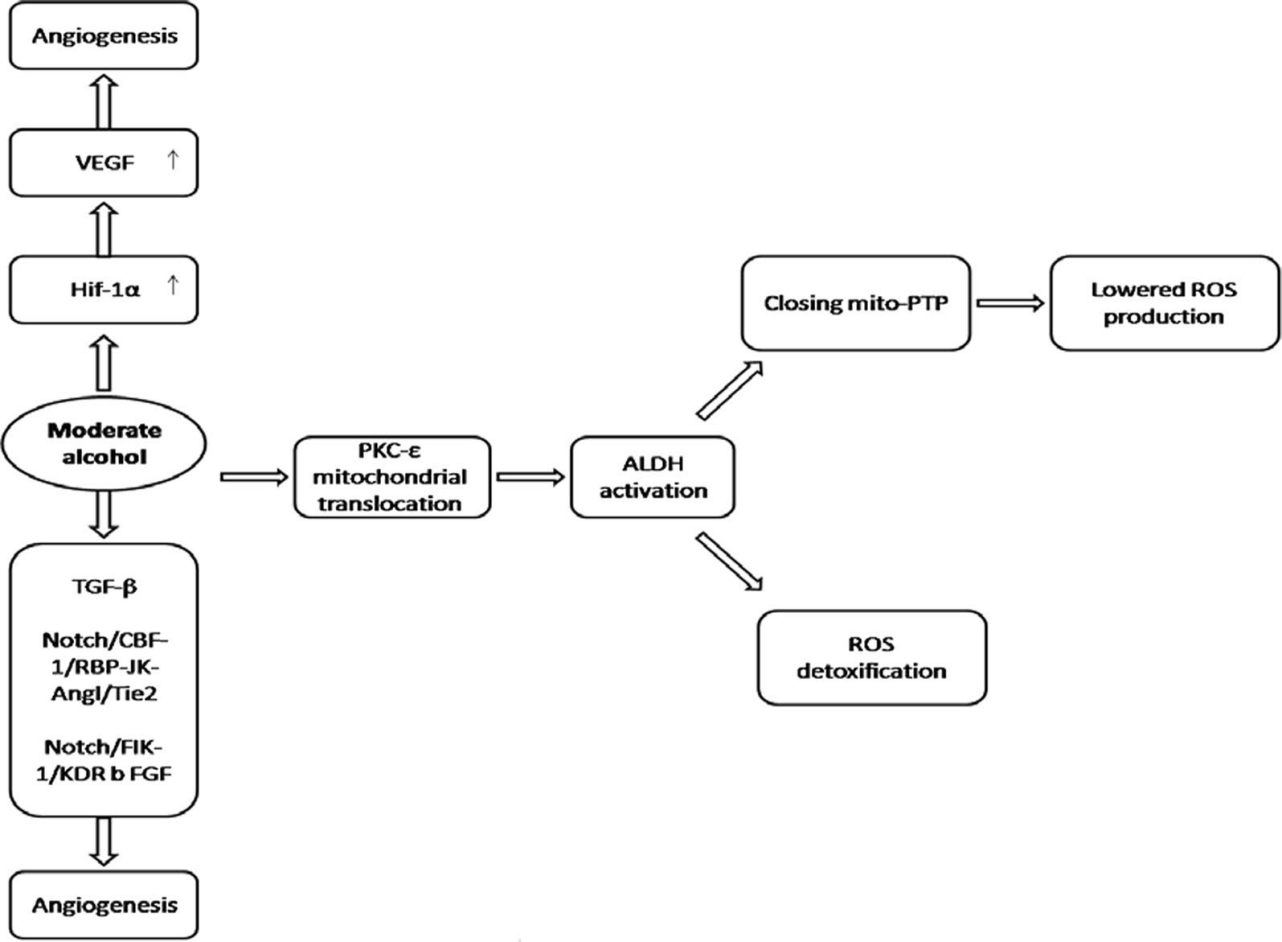
Beneficial effects of moderate alcohol consumption on coronary microcirculation. Positive impact of moderate alcohol consumption or ethanol preconditioning on coronary microvascular integrity and function, protecting against post-MI and PCI microvascular injuries

Angiogenic effects of moderate ethanol were concomitant with other beneficial cardio-protective effects including improved microvascular reactivity, endothelial function and myocardial perfusion in the ischemic regions of the myocardium [120, 121]. Interestingly, in the non-ischemic regions of myocardium distant from the ischemic territory, the blood flow and the endothelial microvascular reactivity showed no significant difference between the alcohol naïve and moderate EtOH group [120]. Moderate levels of ethanol is shown to positively regulate HIF-1α mRNA expression as the transcription factor upstream of VEGF [122]; and the HIF-1α protein is mainly stabilized upon hypoxic conditions while rapidly degraded at normal oxygen levels [123]. This could explain why the beneficial effects of alcohol only occur at ischemic regions, since HIF-1α is expressed, but degraded (Fig. 3). The other suggested mechanisms for the angiogenic effects of moderate ethanol are the induction of pro-angiogenic factors including basic fibroblast growth factor (bFGF), transforming growth factor-β1 (TGF-β1) [124], and Notch/CBF-1/RBP-JK -Ang1/Tie2 or Notch/ Flk-1/KDR pathways in endothelial cells as indicated by in vitro studies [125, 126]. On the other hand, inhibition of angiogenesis by high alcohol supplement may be attributed to the p53 up-regulation following excessive alcohol consumption [127]. P53 is known to have anti-angiogenic features [128]. The other potential mechanisms could be inhibited VEGF signaling as shown to occur in the endothelial cells upon intoxication by high doses of ethanol [129]. It was shown that ethanol inhibits the VEGF signaling in vitro regardless of the level of VEGF expression, via suppressing the phosphorylation and the expression level of VEGF receptors [129].

Moderate chronic ethanol preconditioning was indicated to protect against IRI [130–132], whereas consumption of acute alcohol before IRI does not confer cardioprotection [133, 134].

Proposed mechanisms of alcohol-mediated IRI protection include PKC-ε-ALDH activation, and mitoPTP (mitochondrial permeability transition pore) closing, which reduce the production and release of reactive oxygen species (ROS) [130, 131], as well as the VEGF-induced neovascularization which compensates for the IRI-induced cell death [132]. PKC‐ε activation and its cardiac mitochondrial translocation are triggered by moderate ethanol exposure. Inside mitochondria, it interacts with, and activates ALDH, which plays a critical role in reactive aldehydes detoxification and protection against mitochondrial-originated oxidative stress [130, 131, 135]. In addition, ALDH2 mitochondrial translocation inhibits opening of mitochondrial Permeability Transition Pore (mitoPTP), and thus leads to cardioprotective outcomes [136–138] MPTP is a mitochondrial membrane complex, which opens at highly stressed conditions of the cell (e.g. IRI, endotoxin, and anticancer agents), permitting the flow of the mitochondrial metabolites and ions, which leads to the induction of cell death [138]. Li et al., 2010 found that low concentration ethanol post-conditioning confers cardioprotection against IRI via inhibition of mitoPTP opening, associated with improved hemodynamics and smaller infarct size [136].

Further studies are essential to demonstrate the beneficial or harmful effects of alcohol-induced microvascular alterations, specifically in the setting of different cardiac diseases. Based on the studies available to this date, alcohol-induced microvascular remodeling can be directed toward a beneficial or disadvantageous path depending on the dose and the pattern of alcohol drinking.

## Opioids and coronary microvascular function

### Clinical effect of opioids on coronary microvasculature and CMD

#### Impact of opioids on coronary microvasculature and stable CMD

Many studies have associated opioid abuse with higher risk of CAD [139–152]. Nonetheless, the effect of opium on the coronary microvascular dysfunction (CMD) is under-studied. A cross-sectional study undertaken in a city of Iran with almost 30% rate of opioid addiction in the rural areas [153–155], analyzed stable angina patients, with normal angiography, diagnosed with CMD. The results implicated that opium addiction acts as an independent risk factor in CMD development [155]. In addition to opioid addiction, the effect of opioid-based anaesthetic substances on coronary microcirculation integrity showed microvascular perfusion impairment (long flow recovery times, and slower rate of oxygen re-saturation) [156].

#### Mechanistic ex vivo and in vitro evidence on the effects of opioid on coronary microvasculature

Moreover, experiments in animal models indicated that morphine aggravates the destructive effect of hypertension on coronary microvessels, via inhibition of angiogenesis and lowering the capillary density, as well as by deteriorating endothelial cell function in NO (nitrite oxide) production [157].

### Impact of opioid on post-ischemic and PCI-induced coronary microvascular injury

#### Clinical studies on the impact of opioid on coronary microvasculature injury by reperfused myocardial infarction

Controversial reports on the impact of morphine and opioid agonists on mortality rate and myocardial damage post MI and PCI have been published suggesting a cardioprotective [158–160], adverse effect [161–163] or no significant change [164, 165]. In reperfused STEMI patients, CMR analysis suggested contradictory results by different studies as no impact of morphine on microvascular obstruction (MVO) [165] versus an adverse effect exacerbating the myocardial and microvascular damage (MVO) post PCI [162]. Future clinical trials are warranted to assess the effect of opioids on coronary microvascular function in post MI and Post-PCI patients to determine the safety of using opioid analgesics for pain treatment of ischemic cardiovascular patients.

#### Ex vivo and in vitro effects of opioid on post ischemic and PCI-induced microvascular injury

In vitro treatment of cultured endothelial cells and cardiac myocytes with morphine results in a marked reduction of VEGF expression. Subsequently the reduction of VEGF can lead to the inhibition of the neovascularization and the suppressed re-growth of the capillary network to restore the myocardial perfusion necessary to recover from ischemic injuries [166]. On the other hand, multiple studies suggest a cardioprotective role for opioids, indicating that the administration of opioid agonists such as morphine [167–171], fentanyl [172, 173] and methadone [174] attenuate the ischemic-triggered apoptosis and inflammation following myocardial ischemia, when used as a pre- or post-conditioning agent, or as pain treatment [170, 175–179]. Correspondingly, in vitro and ex vivo studies suggested that the enhanced opioid signaling diminished the cell death induced by ischemia-associated hypoxic injury [175, 180–183]. Summary of the human, animal and in vitro studies on the clinicopathology effects of substance use on the coronary microcirculation are presented in Table 1.

**Table 1:** Summary of the human, animal and in vitro studies on the clinicopathology effects of substance use on the coronary microcirculation

Patient or animal model	Study population	Substance	Administration duration/dose	Clinical test/experiment	Observed effect on coronary microcirculation	References
Young healthy smokers	30	Cigarette Smoking	Short-term chronic	Positron emission tomography measuring myocardial perfusion during rest, cold stress and dipyridamole-induced hyperemia	Impaired myocardial microcirculation function and regulation at cold stress (endothelial-dependent)	[184]
Male healthy twins	360	Cigarette Smoking	Chronic smokers	Positron emission tomography measuring myocardial perfusion at rest and adenosine vasodilation	Lowered CFR in smokers, even after adjusting for oxidative stress and inflammatory markers	[185]
				Inflammation markers: IL-6, CRP		
				Oxidative stress markers: hydroperoxides, GSH,/GSSG ratio		
Healthy young male smokers	30	Cigarette Smoking	Short-term chronic	Positron emission tomography measuring Myocardial blood flow (MBF) at rest, adenosine and cold stress	Reduced ratio of cold MBF to rest MBF (endothelium-dependent)	[186]
Healthy smokers	19	Cigarette Smoking	Chronic smokers	Positron emission tomography measuring coronary flow reserve (CFR), before and after vitamin C administration	Vitamin C restored CFR and the responsiveness of coronary microvessels	[187]
Angina patients, female, no CAD	3568	Cigarette Smoking	Chronic smokers	Doppler echocardiography measuring coronary flow velocity reserve (CFVR) at rest and high dose dipyridamole	Current smoking was identified as a predictor of impaired CFVR	[188]
Vasospastic angina pectoris (VSA) patients	22	Cigarette Smoking	Chronic smokers	Doppler echocardiography measuring coronary flow reserve (CFR), at rest and adenosine administration	Lowers CFR in smokers	[189]
CAD patients	97	Cigarette Smoking	Chronic smokers	Coronary angiography measuring CFR, index of microcirculatory resistance (IMR), and fractional flow reserve (FFR); at rest and adenosine-induced hyperemia	Higher IMR in current smokers, no difference in CFR or FFR	[190]
Healthy young volunteers	20	Cigarette smoking	Chronic and acute effect (chronic smokers with 4 h abstinence, smoking 2 cigarettes)	Doppler echocardiography measuring coronary flow velocity (CFV), and coronary vascular resistance index (CVRI)	No difference in CFR and CVRI at baseline, lower CFR and higher CVRI after smoking 2 cigarettes	[191]
Healthy young volunteers	20	Cigarette smoking	Acute (2 cigarette)	Doppler echocardiography measuring coronary flow reserve (CFR)	Similar reduction in CFR after light and regular cigarette smoking	[192]
Healthy young smokers	62	Cigarette smoking	Chronic and acute effect of light cigarette smoking vs. regular cigarette smoking	Doppler echocardiography measuring coronary flow velocity reserve (CFVR)	Both chronic and acute effects of regular and light cigarettes were similar, reducing the CFVR	[193]
Healthy smokers	21	Cigarette smoking	Acute, cigarettes with either > 1 mg, or < 1 mg nicotine content	Doppler echocardiography measuring coronary flow reserve (CFR)	Reduced CFR only in group smoking > 1 mg content cigarettes	[194]
Healthy smokers	51	Cigarette smoking	Chronic	Measuring plasma and urine biomarkers of inflammation ( IL-6, IL-8, ILβ1 and TNFα), endothelial injury (Intracellular adhesion molecule 1, metalloproteinase-9) and oxidative stress (myeloperoxidase, 8-isoprostane)	Biomarkers of inflammation, oxidative stress, immunity and tissue injury were increased in smokers	[195]
Human coronary arterioles (HCAs)	-	Cigarette Smoking	Chronic smokers	Dissected human coronary arterioles obtained from cardiac surgery; reactivity and responsiveness of microcirculation was tested by video microscopy	Smoking impaired Na^+^/K^+^ ATPase mediated vasodilation	[196]
PCI patients	2765	Cigarette Smoking	Chronic current or past smokers	Health related quality of life(HRQOL) and disease specific health status analyzed by questionnaires	Better cardiac health related outcomes in non-smokers and past smokers, compared to current smokers	[119]
Alcoholic patients expired for advanced liver disease, with no CAD symptoms	18	EtOH	Chronic (alcoholic)	Histology of endomyocardial biopsies	Endothelial cell degeneration, small lumen size, thickened micro-vessel walls with edema, perivascular fibrosis, vascular, subendothelial humps, and vascular wall inflammation	[129]
Rat model	21 animals in each test group	EtOH	Chronic, 36% ethanol containing diet (4 weeks)	Histology	Thickened walls of micro-vessels and smaller lumen size, increased endothelial proliferation	[121]
Rabbit model	10 animals per group	EtOH	Chronic ( diet containing 20% ethanol) 3 weeks	Histology and ultra-structural analysis of the myocardium and cardiac capillaries	Increased numerical density of the micro-vessels	[120]
Alcoholic patients	40	EtOH	Chronic (alcoholic patients)	Histopathology analysis on cardiac biopsies obtained	Increased capillary density with enhanced endothelial proliferation	[122]
C57BL/6 J mice	7 animals per group	EtOH	Chronic (diet containing 36% ethanol) 12 weeks	Histology	Remodeling of the microcirculation, capillaries with widened peri-capillary distances	[125]
Rats preconditioned before myocardial infarction induction	8 animals per group	EtOH	Chronic (preconditioned with low-dose ethanol (0.5 g/kg/day), high-dose ethanol (5 g/kg/day) of alcohol 4 weeks before MI induction	Immunohistochemistry	High dose: endostatin increased, no change in VEGF	[126]
					Low dose: increased VEGF, lowered endostatin	
Cultured small-vessel murine endothelial cells 4–10 (SVEC4-10)	-	EtOH	Acute, 100 mg/dl	In vitro angiogenesis assay, Endothelial cell tube formation assay	Impaired angiogenesis and reduction in VEGF receptors	[127]
Yorkshire swine	14	EtOH	Chronic 7 weeks after MI induction, 90 ml ethanol daily,	Dissected micro-vessels /vasodilator response and histopathology analysis	Increased angiogenesis, improved microvascular reactivity, endothelial function and myocardial perfusion in the ischemic regions of the myocardium	[155]
Yorkshire swine	16	EtOH	Chronic, 90 ml ethanol daily, 7 weeks	Dissected micro-vessels /vasodilator response and histopathology analysis	Increased capillary density, increased VEGF, no change in microvessel reactivity and myocardial perfusion	[156]
Coronary artery vascular smooth muscle cells		EtOH	Acute, 10–20 mM	protein and mRNA analysis	Increased VEGF	[166]
Human umbilical vein endothelial cells (HUVECs)		EtOH	Acute, 24 h, 1–100 mM	Matrigel network formation assay, proliferation and migration assay, protein and mRNA analysis	Activation of CBF-1/RBP-Jk mediated angiogenesis	[23]
Human coronary artery endothelial cells (HCAECs)	-	EtOH	Acute, 24 h, 1–50 mM	Matrigel network formation assay, protein and mRNA analysis	Activation of Flk-1/Notch mediated angiogenesis	[24]
Rat model	26	EtOH	Chronic, 3–24 month age, 12% in drinking water	mRNA extraction from left ventricles of the heart, qRT-PCR	Higher expression of p53	[23]
Patients with chest pain, positive ETT, normal angiography	250	Opium	Chronic (addicted patients)	Coronary angiography	Opium addicted patients are more likely to develop CMD	[24]
Patients with CAD, scheduled to undergo coronary artery bypass grafting surgery	35	Opioid-based anesthetic (fentanyl)	Acute anesthetic dose of fentanyl up to 5 mg kg-1, 30 min)	Vascular occlusion testing (VOT) and near-infrared spectroscopy	Impaired coronary microvascular reactivity	[156]
Cultured cells: mouse endothelial cells and cardiac myocytes	-	Morphine	1–100 ng/ml	Biomolecular tests for analysis of VEGF expression (qPCR, ELISA)	Reduced VEGF expression by cardiomyocytes and endothelial cells	[166]

## Conclusion

Overall, the presented evidence points to the importance of considering smoking, excessive alcohol use, and opioid addiction as independent risk factors in the development of the coronary microvascular disease. Moreover, evidence supports a cardioprotective role for moderate ethanol and opioids particularly against post-ischemic and intervention-mediated injuries to the coronary microcirculation. Almost 80% of the coronary vascular resistance is due to the microcirculation [23, 24]. Therefore the dysfunction of coronary microcirculatory network (CMD disease) can mainly impair the coronary blood perfusion, which results in cardiac damage [23, 24]. CMD has become a subject of attention among cardiologists and researchers over the last thirty years. Thus far, the studies have led to the identification of some of the mechanisms underlying the structural and functional impairment in the coronary microvasculature. Nevertheless, this is still a growing field and requires much further investigations. As of today, the diagnosis of CMD is still facing complications and misdiagnosis due to the technical limitations in imaging the coronary microcirculation, and the high cost and the invasiveness of the available clinical technologies. Therefore, more attention needs to be directed toward discovery of peripheral diagnostic markers for CMD, which are lacking.

Overall, more ultra-structural, molecular and histopathology assessments of the differential effects of various risk factors of CMD including smoking, alcohol, and drug addiction are essential. Combined with application of the most advanced imaging techniques for myocardial capillary network, future studies could lead to the development of novel specific diagnostic markers and therapeutic strategies for CMD, as well as preparation of accurate administration protocols for substances such as ethanol and opioid in preconditioning for ischemic cardiac disease in clinic.

## Data Availability

All data used for this review study are available within the manuscript.

## Abbreviations

CVD: Cardiovascular diseases
AMI: Acute myocardial infarction
CAD: Coronary artery disease
CMD: Coronary microvascular dysfunction
CFR: Coronary flow reserve
CVRI: Coronary vascular resistance index
PCI: Percutaneous coronary intervention
VEGF: Vascular endothelial growth factor
IRI: Ischemic reperfusion injury
MVO: Microvascular intraluminal obstruction
mitoPTP: Mitochondrial permeability transition pore
